# Interfacial Engineering of High-Performance Pickering Emulsion–Gelatin Composite Films for Active Packaging

**DOI:** 10.3390/foods14223978

**Published:** 2025-11-20

**Authors:** Jia Kan, Mingzhu Li, Menghuan Liu, Ning Jiang, Zefeng Yue, Hao Yu, Rongxue Sun, Qianyuan Liu, Saikun Pan, Cheng Wang

**Affiliations:** 1 Institute of Agricultural Products Processing, Jiangsu Academy of Agricultural Sciences, Nanjing 210014, China; 17305263586@163.com (J.K.); m19741895219@163.com (M.L.); 17397495371@163.com (M.L.); yzf6782000@163.com (Z.Y.); 17855530610@163.com (H.Y.); sunrongxue187@163.com (R.S.); liu.qianyuan@foxmail.com (Q.L.); 2 Integrated Scientific Research Base for Preservation, Storage and Processing Technology of Aquatic Products of the Ministry of Agriculture and Rural Affairs, Nanjing 210014, China; 3 College of Marine Food and Bioengineering, Jiangsu Ocean University, Lianyungang 222000, China; pskgx@163.com

**Keywords:** gelatin, Pickering emulsion, interfacial interactions, active packaging

## Abstract

Amidst the urgent demand for sustainable alternatives to petrochemical plastics, this work incorporated oregano essential oil Pickering emulsion (AOPE; stabilizer: acetylated chitin nanocrystals (a-ChNCs)) into the gelatin matrix. Through precisely engineered hydrogen-bonding networks at the a-ChNCs/gelatin interface, achieved through the systematic optimization of AOPE concentration, a high-performance bio-based gelatin composite film (designated as GOP_X%_) was developed. Low-field nuclear magnetic resonance analysis confirmed that GOP_X%_ containing AOPE exhibited increased hydrogen bonding crosslink density. At an AOPE loading of 6% (GOP_6%_), the composite film exhibited exceptional improvements compared with GOP_0%_: elongation at break increased by 107%, toughness increased by 167.5%, water vapor permeability decreased by 73.6%, and oxygen permeability reduced by 85.3%. Additionally, antibacterial and antioxidant properties were markedly enhanced. The Pickering emulsion effectively mitigated the damage of ultraviolet radiation and thermal effects on the bioactive properties of oregano essential oil. Overall, the incorporation of AOPE imparted the gelatin composite film with exceptional mechanical properties, barrier properties, antioxidant activity, and antibacterial activity, extending the shelf life of grass carp fillets by 3 days during storage. This sustainable and eco-friendly active packaging film offers a promising strategy for designing active packaging materials.

## 1. Introduction

Petroleum-derived plastic packaging materials are extensively employed in the food industry due to their superior properties and economical cost. Nevertheless, their resistance to biodegradation gives rise to severe environmental issues. Over time, they can break down into microplastics, which may enter the food chain and pose potential health risks to humans [1]. Biobased packaging materials (e.g., gelatin, sodium alginate, starch, and chitosan) have emerged as viable alternatives to conventional petroleum-derived plastics due to their renewable, biodegradable, and non-toxic properties. Moreover, these biopolymers can be engineered to incorporate bioactive agents, thereby acquiring functional properties such as antioxidant, antibacterial, and anticorrosive abilities. These characteristics make them well-suited to meet the diverse needs of the food industry [2].

In recent years, to enhance the antibacterial and antioxidant properties of biobased biodegradable packaging films, natural active preservatives (such as essential oils, polyphenols, flavonoids, lysozyme, and ε-polylysine) have commonly been incorporated into the biobased polymer matrix [3]. Among them, essential oils (EOs) have gained significant attention as natural food preservatives due to their strong antibacterial and antioxidant properties [4]. They have been approved by the Food and Drug Administration (FDA) for use in food [5]. Specifically, oregano essential oil (OEO), extracted from Origanum vulgare of the Labiatae family, is recognized as a high-quality natural bioactive agent. Its effectiveness is primarily attributed to active compounds such as carvacrol and thymol, which demonstrate remarkable antibacterial and antioxidant properties, making OEO highly valuable in food preservation [6]. The application of EOs in active packaging films faces several limitations, including their high volatility, thermal instability, and low water solubility [7]. Furthermore, the intrinsic hydrophobicity of EOs often leads to incompatibility with hydrophilic polymer matrices, resulting in phase separation that diminishes the mechanical strength and barrier properties of packaging films [8]. Liu et al. incorporated lavender essential oil into soluble soybean polysaccharide/pullulan blend films, enhancing their antibacterial and antioxidant activities. Nevertheless, this incorporation resulted in non-uniform, discontinuous microstructures that reduced the film’s mechanical strength [9]. Chen and Liu further elucidated that the reduced barrier properties of cellulose sulphate films following EO incorporation primarily stems from two mechanisms [10]: (1) during the film drying, the high volatility of EOs facilitates pore formation, thereby extending the diffusion pathways for water vapor molecules; (2) EOs disrupt the inherent uniform and continuous polymer network, potentially increasing water vapor transmission. Consequently, developing effective strategies to integrate EOs into bio-based packaging films, while maintaining their mechanical and barrier properties as well as their antibacterial and antioxidant activities, holds significant scientific and practical importance.

Pickering emulsification has drawn increasing interest as an eco-friendly and efficient approach for stabilizing emulsions and encapsulating active compounds [11]. This method relies on the interfacial adsorption of amphiphilic solid particles to reduce interfacial tension, thereby forming a stable protective layer around droplet surfaces. Such particle-based stabilization offers distinct advantages over conventional encapsulation systems, including surfactant-stabilized emulsions and lipid-based liposomes. Due to the irreversible adsorption of solid particles, Pickering emulsions create a rigid interfacial barrier that markedly enhances kinetic stability against coalescence and Ostwald ripening [12]. Moreover, this method eliminates the need for synthetic surfactants, improving both biocompatibility and safety. The rigid particle shell provides enhanced thermal protection for the encapsulated core compared with the lipid bilayers of liposomes [13]. This technique utilizes the adsorption of amphiphilic solid particles to reduce interfacial tension, thereby forming a stable protective layer on droplet surfaces. The formation of this protective layer effectively prevents the volatility of essential oils and the degradation of their activity, while also mitigating the issue of structural discontinuity observed when essential oils are directly incorporated into films [14]. Chitin, notable for its renewability, biodegradability, and abundant availability, has become a focal point of research [15]. Chitin-derived chitin nanocrystals (ChNCs) are rod-like polysaccharide nanomaterials with high aspect ratios, which can be incorporated into films to markedly enhance mechanical properties and thermal stability while reducing water absorption and solubility [16]. Although ChNCs can be used as Pickering emulsion stabilizers, their high surface hydroxyl density and inherent hydrophilicity often result in reduced emulsion stability [17].

In this study, acetylated chitin nanocrystals (a-ChNCs) were prepared through acetylation modification. Owing to their higher surface charge level and amphiphilicity, a-ChNCs exhibited significantly enhanced dispersion and stability in aqueous suspensions. Pickering emulsions were then prepared using OEO as the oil phase and a-ChNCs as the stabilizer. By incorporating these Pickering emulsions into gelatin-based films, gelatin composite films (GOP_X%_) were constructed via interfacial interactions between a-ChNCs and gelatin molecules. The following beneficial effects were observed: firstly, a-ChNCs not only effectively stabilized the Pickering emulsions but also acted as nanofillers in the films, which contributed to the enhanced mechanical and barrier properties of the films; secondly, the presence of OEO in the emulsion endowed the composite films with antibacterial and antioxidant properties. Fourier transform infrared spectroscopy (FTIR), X-ray diffractometry (XRD), and Low-field nuclear magnetic resonance (LF-NMR) confirmed the existence of interfacial interactions (hydrogen bonds). A comprehensive evaluation of the composite films was systematically assessed, covering their mechanical and barrier properties, UV resistance, antioxidant and antibacterial activities, along with their release characteristics. To explore the practical application potential of these composite films in food preservation, they were applied to the preservation of grass carp fillets. Measurements of the physicochemical indexes and texture changes in the grass carp fillets provided evidence that the gelatin composite films could effectively extend the shelf life of food products.

## 2. Materials and Methods

### 2.1. Materials

Chitin (M_W_: 203.19, ≥95%), Tris base (≥99%), 5,5′-Disulfanediylbis (2-nitrobenzoic acid) (≥98%) and gelatin (GEL, gel strength: 250 g, Bloom, ≥98%) were purchased from Shanghai Aladdin Biochemical Technology Co. (Shanghai, China). Acetic anhydride (≥98.5%) and sulfuric acid (≥98%) were purchased from Sinopharm Chemical Reagent Co., Ltd. (Shanghai, China). Oregano essential oil (OEO, ≥98%, carvacrol (85.0 ± 1.2%), thymol (1.1 ± 0.1%)), Folin–Ciocalteu’s phenol reagent (BR) were purchased from Shanghai Yuanye Bio-Technology Co., Ltd. (Shanghai, China). Glycerol (≥99%), potassium persulfate (≥99.9%), trichloroacetic acid (TCA, ≥99%), magnesium oxide (≥99%), 2,4-dinitrophenylhydrazine (≥98%), 2-thiobarbituric acid reagent (TBA, ≥98%), ethylenediaminetetraacetic acid disodium salt (≥99%), methylene blue (≥98%), bromophenol blue (BR) and methyl red (≥95%) were purchased from Shanghai Macklin Biochemical Technology Co., Ltd. (Shanghai, China). *Escherichia coli* (*E. coli*, ATCC25922) and *Staphylococcus aureus* (*S. aureus*, ATCC25933) were provided by Jiangsu Academy of Agricultural Sciences (Nanjing, China).

### 2.2. Preparation of Acetylated Chitin Nanocrystals (a-ChNCs)

The acetylated chitin nanocrystals (a-ChNCs) were prepared through a two-step process modified from a reported method [18]. First, chitin nanocrystals (ChNCs) were obtained by dispersing 3 g of chitin in 100 mL of 3 M H_2_SO_4_ and hydrolyzing at 90 °C under magnetic stirring (200 rpm) for 12 h. The reaction was quenched by tenfold dilution with distilled water and stored at 4 °C overnight. The supernatant was removed, and the precipitate was centrifuged (10,000 rpm, 10 min, three times), dialyzed against ultrapure water for 3 d to reach a constant pH, and ultrasonicated (500 W, 1 h) to yield a ChNC suspension. Subsequently, the ChNCs suspension was centrifuged and washed twice with anhydrous ethanol to remove residual water. The ChNCs were redispersed in glacial acetic acid and sonicated for 10 min. Acetic anhydride was then added dropwise at a 5:1 mass ratio (acetic anhydride: ChNCs) in the presence of methanesulfonic acid catalyst. The reaction was maintained at 45 °C for 4 h, followed by washing three times with anhydrous ethanol and repeatedly with deionized water until neutral. The final product was re-sonicated and dispersed in deionized water to obtain a stable a-ChNCs suspension.

The chemical structures of ChNCs and a-ChNCs were characterized by Fourier transform infrared spectroscopy (FTIR, Tensor II, Bruker, Ettlingen, Germany) in the range of 600–4000 cm^−1^ with 64 scans at a resolution of 2 cm^−1^. The morphology of a-ChNCs was examined using transmission electron microscopy (TEM, HT7800, Hitachi, Tokyo, Japan) operated at an accelerating voltage of 120 kV. The hydrodynamic diameter and zeta potential of ChNCs and a-ChNCs were measured by dynamic light scattering (DLS, Zetasizer Nano ZS, Malvern, Worcestershire, UK). The crystalline structure was analyzed by X-ray diffraction (XRD, SmartLab, Rigaku, Tokyo, Japan) using Cu Kα radiation (40 kV, 40 mA) over a 2θ range of 5–40° at a scanning rate of 5°/min. The crystallinity index (*X_c_*) of a-ChNC was calculated according to Equation (1) [19]:

(1) $$X_{c}\;(\%)\;=\;\frac{I_{110}\;-\;I_{\mathrm{am}}}{I_{110}}\;\times \;100\%$$

where *I*_110_ is the maximum intensity of the (110) lattice diffraction (expressed in arbitrary units) and *I*_am_ is the minimum intensity representing the amorphous background, typically observed around 2θ = 16°.

### 2.3. a-ChNC-Stabilized Oregano Essential Oil (OEO) Pickering Emulsions (AOPE)

Pickering emulsions were prepared according to the previously reported method [20]. The emulsion was prepared by mixing 2 g of OEO with 8 g of the 1% (*w*/*v*) aqueous a-ChNC dispersion (OEO: a-ChNCs dispersion ratio = 1:4, *w*/*w*). The mixture was homogenized using a high-speed disperser at 12,000 rpm for 5 min, followed by ultrasonic treatment at 350 W for 5 min under ice bath conditions.

The particle size distribution and polydispersity index (PDI) of the emulsions were determined by DLS using a Malvern Zetasizer Nano ZS (Malvern Instruments, Worcestershire, UK) at 25 °C. AOPE appearance was photographed during different storage times. The micromorphology of AOPE was directly observed using an optical microscope (ML31, Mshot, Guangzhou, China). Before observation, the emulsion was diluted 20-fold with deionized water, and 20 μL of the diluted sample was placed on a clean glass slide for microscopic imaging. The encapsulation rate (EE) of the emulsion was determined based on the method described by Almasi et al. [21], with minor modifications. Briefly, 0.5 mL of the emulsion and 1 mL of n-hexane were placed into 2 mL centrifuge tubes. After centrifugation at 8000 rpm for 10 min, the supernatant was collected and diluted 1000-fold. The absorbance at 276 nm was then measured, and the concentration of unencapsulated essential oil was calculated using the standard curve equation (Figure S1). The encapsulation rate (EE) was calculated using the following equation:

(2) $$\mathrm{EE}\;(\%)\;=\;\frac{C_{0}\;-\;C_{1}}{C_{0}}\;\times \;100\%$$

where C_0_ is the total concentration of OEO used in the emulsion (mg), and C_1_ is the concentration of unencapsulated (free) OEO (mg).

### 2.4. Preparation of Gelatin/AOPE Composite Films (GOP_X%_)

4 g of gelatin was dissolved in 100 mL of distilled water and hydrated at 25 °C for 30 min. The solution was then heated to 60 °C while stirring for 30 min. After cooling to 45 °C, 1.2 g of glycerol was added and the mixture was stirred for another 30 min to achieve homogeneity. AOPE was incorporated at different amounts (0, 0.5, 1, 1.5, and 2 g) into 25 g of gelatin solution, yielding films designated as GOP_0%_, GOP_2%_, GOP_4%_, GOP_6%_, and GOP_8%_, according to the AOPE content. The mixture was homogenized at 15,000 rpm for 4 min using a high-speed homogenizer, followed by vacuum defoaming and drying at 35 °C for 24 h. Following the drying process, the films were equilibrated at 25 °C and 60% RH for 48 h prior to undergoing further testing.

### 2.5. Characterization of GOP_X%_

The microstructure (surface and cross-section) of the composite film was observed using a scanning electron microscope (SEM, Regulus 8100, HITACHI, Tokyo, Japan). The chemical structure of the composite film was analyzed using a Fourier transform infrared (FTIR) spectrometer (Tensor II, Bruker, Ettlingen, Germany) in the range of 600–4000 cm^−1^, with a total of 64 scans at a resolution of 2 cm^−1^. Crystalline structures were characterized using an X-ray diffractometer (D2 Phaser, Bruker, Ettlingen, Germany) with Cu Kα radiation at 40 kV and 30 mA, scanning from 5 to 60° at a rate of 5°/min. Thermogravimetric analysis (TGA) was performed using a thermogravimetric analyzer (SDT650, TA Instruments, New Castle, DE, USA) under a nitrogen atmosphere, with samples heated at 10 °C/min.

### 2.6. Mechanical Properties

The mechanical properties of the composite film were evaluated by a universal testing machine (AGS-X, Shimadzu, Kyoto, Japan) following the ASTM D882-22 standard [22]. Tests were conducted at 25 ± 2 °C and 60 ± 5% RH, with a strain rate of 50 mm/min.

### 2.7. UV–Visible Transmittance

The transmittance of the composite film was measured using a UV-Vis spectrophotometer (E220, Thermo Scientific, Waltham, MA, USA) over the wavelength range of 200–800 nm. The blocking percentages for UVC (200–280 nm), UVB (280–320 nm), and UVA (320–400 nm) were calculated by Equations (3)–(5), respectively:

(3) $$\mathrm{UVC}\;\mathrm{Blocking}\;=\;1-\frac{\int_{200}^{280}T\left(\varphi \right)}{\int_{200}^{280}d\left(\varphi \right)}\;\times \;100\%$$

where T(φ) is the average transmittance of the composite film and d(φ) is the bandwidth for the UVC range.

(4) $$\mathrm{UVB}\;\mathrm{Blocking}=1-\frac{\int_{280}^{320}T\left(\varphi \right)}{\int_{280}^{320}d\left(\varphi \right)}\;\times \;100\%$$

where T(φ) is the average transmittance of the composite film and d(φ) is the bandwidth for the UVB range.

(5) $$\mathrm{UVA}\;\mathrm{Blocking}=1-\frac{\int_{320}^{400}T\left(\varphi \right)}{\int_{320}^{400}d\left(\varphi \right)}\;\times \;100\%$$

where T(φ) is the average transmittance of the composite film and d(φ) is the bandwidth for the UVA range.

### 2.8. Water Vapour Permeability (WVP) and Oxygen Permeability (OP)

The WVP values of GOP_X%_ films were measured by a water vapor transmission rate test system (C390H, Labthink Instruments Co., Ltd., Jinan, China). Measurements were conducted in triplicate at 25 °C and 90% RH, with mean values reported [23].

The OP values of GOP_X%_ films were assessed at 25 °C and 50% RH by an oxygen transmission rate system (C230H, Labthink Instruments Co., Ltd., Jinan, China). Measurements were conducted in triplicate, with mean values reported [24].

### 2.9. Swelling Rate (SR) and Water Solubility (WS)

The SR and WS of GOP_X%_ films were determined following the method reported by Sucheta et al. [25]. Film samples (2 cm × 2 cm × 0.08 mm) were initially weighed (W_0_) and dried at 105 °C to constant weight (W_1_). The dried films were immersed in 20 mL of distilled water for 1 h, blotted dry with filter paper, and weighed again (W_2_). The swollen films were then re-immersed for 24 h and dried to a constant weight (W_3_). SR and WS were calculated using the following equations:

(6) $$SR\;(\%)\;=\;\frac{W_{2}-{\;W}_{1}}{W_{1}}\;\times \;100\%$$

(7) $$\mathrm{WS}\;(\%)=\frac{W_{1}-{\;W}_{3}}{W_{1}}\;\times \;100\%$$

### 2.10. Determination of Total Phenol Content (TPC)

The TPC of the composite film was determined using the Folin–Ciocalteu method [26]. Briefly, films (50 mg) were immersed in 10 mL of distilled water overnight. The Folin–Ciocalteu reagent solution was prepared by mixing 0.5 mL of the Folin–Ciocalteu reagent with 7 mL of distilled water. Subsequently, 0.1 mL of the film extract was mixed with 1.5 mL of the Folin–Ciocalteu solution, 0.9 mL of distilled water, and 0.5 mL of 0.1 M Na_2_CO_3_. The mixture was vortexed for 1 min and incubated in the dark at room temperature for 2 h. Absorbance was measured at 765 nm using a UV-Vis spectrophotometer. TPC was calculated based on a gallic acid standard curve (Figure S2), and the results were expressed as mg of gallic acid equivalents per gram of film (mg GAE/g).

### 2.11. Antioxidant Activity

The antioxidant activity of the films was determined using DPPH and ABTS radical scavenging assays. For the DPPH assay, the test was conducted according to a previously reported method with slight modifications [27]. Briefly, 7.88 mg of DPPH powder was dissolved in 100 mL of absolute ethanol to prepare a 0.2 mM DPPH solution. Then, 5 mg of the film sample was mixed with 1.5 mL of the DPPH solution and incubated in the dark at 23 °C for 30 min. After incubation, the supernatant was collected, and its absorbance was measured at 517 nm. The DPPH radical scavenging activity was calculated using Equation (8):

(8) $$\mathrm{DPPH}\;\mathrm{scavenging}\;\mathrm{activity}\;(\%)\;=\;\frac{A_{0}-{(A}_{s}-A_{i})}{A_{0}}\;\times \;100\%$$

where A_0_ is the absorbance of the control, A_s_ is the absorbance of the sample, and A_i_ is the absorbance of the sample measured under identical conditions as A_s_, using ethanol instead of DPPH solution.

The ABTS assay was performed following a reported procedure [26]. ABTS cation radicals were generated by mixing 7 mM ABTS with 2.45 mM potassium persulfate, followed by incubation in the dark at room temperature for 16 h. Before use, the resulting ABTS cation radical solution was diluted with ethanol to achieve an absorbance of 0.7 ± 0.02 at 734 nm. Film samples (0.1 g) were immersed in 20 mL of distilled water and stirred for 12 h. The mixture was centrifuged at 5000 rpm for 5 min, and the supernatant was collected. A 0.5 mL of the supernatant was mixed with 3.5 mL of ABTS radical solution and the absorbance was measured at 734 nm after 30 min of reaction in the dark. A mixture of 0.5 mL distilled water and 3.5 mL ABTS radical solution served as the control. The radical scavenging activity was calculated using the following equation:

(9) $$\mathrm{Radical}\;\mathrm{scavenging}\;\mathrm{activity}\;(\%)\;=\;\frac{A_{\mathrm{control}}-A_{\mathrm{sample}}}{A_{\mathrm{control}}}\;\times \;100\%$$

where A_control_ and A_sample_ represent the absorbance of the control and the sample, respectively.

To evaluate the protective effect of emulsion encapsulation on OEO, the antioxidant activity of GOP_6%_ films was assessed under UV irradiation (exposed under the UV lamp (365 nm, 20 W) for 1, 2 and 3 h, respectively) and heat treatment (55 °C for 1, 2 and 3 h, respectively) [28]. The gelatin film containing the same amount of unencapsulated OEO was designed as the control sample.

### 2.12. Antibacterial Activity

The antibacterial activity of GOP_X%_ films against *E. coli* and *S. aureus* was evaluated using the agar diffusion method [29]. A 0.1 mL sample of bacterial suspension (10^7^ CFU/mL) was uniformly spread onto Luria–Bertani (LB) agar plates. Wells (6 mm diameter) were created using a sterile punch, and 100 µL of the film-forming solution was added to each well. The plates were incubated at 37 °C for 12 h, after which the diameters of the inhibition zones were measured.

### 2.13. Release Characteristics of GOP_X%_

The release behavior of OEO from the composite film was evaluated at 25 °C using 10% and 50% ethanol as food simulants [30]. Film samples (100 mg) were cut into pieces (16 mm × 4 mm × 0.08 mm) and immersed in 10 mL of each simulant. At predetermined intervals, 1 mL of the solution was withdrawn for absorbance measurement at 274 nm using a UV-Vis spectrophotometer, and an equal volume of simulant was added to maintain constant volume. The released OEO was expressed as μg per 100 mg of film.

The release kinetics of OEO from the films were evaluated using two kinetic models, namely the Higuchi model (*M_t_/M_∞_* = *k × t*^1/2^) and the Korsmeyer–Peppas model (*M_t_/M_∞_* = *k × tⁿ*) [31]. Here, *M_t_* is the amount of OEO released at time t, *M_∞_* is the total amount released, *k* is the release rate constant, and *n* is the release exponent.

### 2.14. Applications in Fish Preservation

The GOP_6%_ film exhibited the best overall performance, with high tensile strength and elongation, while GOP_8%_ showed micropores under SEM, reducing mechanical and barrier properties. Considering efficient OEO utilization and minimal dosage, GOP_6%_ was identified as the optimal formulation, providing both robust material properties and effective antimicrobial and antioxidant activity for food preservation.

Fresh grass carp were purchased from a local market, where they were slaughtered on-site by the sellers using standard humane slaughter procedures to minimize stress. The samples were placed on ice and transported to the laboratory within 30 min for subsequent processing. Grass carp were cut into uniform samples (3 cm × 3 cm × 2 cm; ~20 g) rinsed with sterile water, and randomly assigned to four experimental groups: PE-wrapped (PE), GOP_0%_-wrapped (GOP_0%_), GOP_6%_-wrapped (GOP_6%_), and unwrapped controls (CK). All samples were stored at 4 °C and analyzed at 3-day intervals for preservation indicators.

#### 2.14.1. Determination of pH and Total Volatile Basic Nitrogen (TVB-N)

A 5 g portion of each sample was homogenized with 50 mL of distilled water for 5 min. The supernatant was then collected via centrifugation, and its pH was measured by a pH meter (FE28, METTLER TOLEDO, Greifensee, Switzerland). The TVB-N values of grass carp fillets were quantified by an automatic Kjeldahl nitrogen analyzer (Kjeltec 8400, Foss, Seattle, WA, USA), with results expressed as mg/100 g of sample.

#### 2.14.2. Determination of Total Viable Count (TVC)

A 5 g sample was homogenized with 45 mL sterile saline for 5 min. The homogenate was then serially diluted with sterile saline solution to achieve an appropriate concentration and then uniformly plated on agar plates. After incubation at 37 °C for 48 h, colony counts were recorded [32].

#### 2.14.3. Determination of Thiobarbituric Acid-Reactive Substances (TBARS)

Determination of TBARS values was performed following a method previously reported by us [33]. A 4 g sample was mixed with 40 mL of 10% (*w*/*v*) TCA solution, homogenized, and shaken at 50 °C for 30 min. After cooling to 25 °C, the mixture was filtered. The filtrate was mixed with 0.02 mol/L TBARS solution in a 1:1 (*v*/*v*) ratio and heated in a water bath at 90 °C for 30 min. Absorbance was then measured at 532 nm, and the malondialdehyde (MDA) content was calculated using the standard curve (Figure S3). TBARS values were expressed as mg MDA/kg of sample.

#### 2.14.4. Texture Profile Analysis (TPA)

Grass carp were cut into 2 cm × 2 cm × 2 cm cubes and kept on ice prior to analysis. Texture profile analysis was performed perpendicular to the muscle fibers using a texture analyzer (TMS-Pro, FTC, Washington, DC, USA) equipped with a P5 probe. Samples were compressed to 50% of their original height at a speed of 1 mm/s. Hardness (N), springiness (mm), gumminess (N), and chewiness (mJ) were derived from the TPA curves [34].

#### 2.14.5. Myofibril Protein Degradation

Myofibrillar protein (MP) was extracted according to a previously reported method with minor modifications [35]. Briefly, 2 g of fish muscle was homogenized with 20 mL of buffer A (0.05 M KCl, Tris-maleate, pH 7.0) and centrifuged at 10,000× *g* for 15 min at 4 °C. This step was repeated twice, and the resulting precipitate was resuspended in 20 mL of buffer B (0.6 M KCl, 20 mM Tris-maleate, pH 7.0), homogenized, and incubated on ice for 3 h. The supernatant containing MP was then collected by centrifugation under the same conditions, and protein concentration was determined using the BCA method.

The total sulfhydryl (T-SH) content was determined as described previously [36]. Ellman’s reagent was prepared as a 20 mM solution in buffer I (0.086 M Tris, 0.09 M glycine, 4 mM EDTA, pH 8.0). Myofibrillar protein (1 mg/mL) was mixed with an equal volume of buffer II (buffer A containing 8 M urea) and centrifuged at 9600× *g* for 15 min at 4 °C. Then, 1 mL of the supernatant was incubated with 20 μL of DTNB at room temperature in the dark for 1 h. Absorbance was measured at 412 nm, and T-SH content was calculated using Equation (10):

(10) $$\mathrm{Sulfhydryl}\;\mathrm{content}\;=\;75.53\;\times {\;A}_{412}/C$$

where A_412_ is the absorbance of the sample at 412 nm; C is the protein concentration (mg/mL); and 75.53 is the converted molar extinction coefficient of Ellman’s reagent.

Carbonyl content was determined via 2,4-dinitrophenylhydrazine (DNPH) derivatization [37]. Briefly, 1 mL of MP solution (2 mg/mL) was mixed with 1 mL of 10 mM DNPH in 2 M HCl and incubated in the dark at room temperature for 1 h. The mixture was precipitated with 20% (*w*/*v*) TCA and centrifuged at 10,000× *g* for 5 min. The pellet was washed three times with ethanol/ethyl acetate (1:1, *v*/*v*), dissolved in 3 mL of 6 M guanidine hydrochloride (2 M HCl), and incubated at 37 °C for 20 min. After centrifugation, the absorbance of the supernatant was measured at 370 nm, and carbonyl content was calculated using Equation (11):

(11) $$\mathrm{Carbonyl}\;\mathrm{content}\;(\mathrm{nmol}/\mathrm{mg})\;=\;A_{370}\;\times \;10^{6}/22,000$$

where A_370_ refers to the experimental group’s absorbance measurement; 10^6^ represents the conversion factor; and 22,000 is the molar extinction coefficient (M^−1^ cm^−1^).

Surface hydrophobicity (H_0_) was determined via a bromophenol blue (BPB) binding assay with minor modifications [38]. Briefly, 40 μL of 1 mg/mL BPB solution was added to 1 mL of MP solution, with the blank consisting of 40 μL BPB mixed with 1 mL distilled water. Samples were incubated at room temperature for 30 min and centrifuged at 10,000× *g* for 5 min at 4 °C. The absorbance of supernatants was measured at 595 nm using a multimode microplate reader (Spark, Tecan, Männedorf, Switzerland), and H_0_ was calculated using Equation (12):

(12) $$\mathrm{BPB}\;\mathrm{bound}\;(\mu g)\;=\;\frac{{40\;\mu g\;\times \;(A}_{2}-A_{1})}{A_{2}}$$

MP solutions were diluted to 1 mg/mL in buffer B (0.6 M KCl, 20 mM Tris-maleate, pH 7.0) and centrifuged at 10,000× *g* for 5 min at 4 °C to remove insoluble aggregates, and the supernatant was used for measurement. Changes in the tertiary structure of MP were evaluated by measuring endogenous fluorescence spectra using a fluorescence spectrophotometer (Cary Eclipse G9800A, Agilent Technologies, Santa Clara, CA, USA). Excitation was set at 280 nm, and emission spectra were recorded from 310 to 420 nm with a 5 nm slit width and a scanning speed of 1200 nm/min [39].

### 2.15. Statistical Analysis

Origin 2024 was used for graphing, and SPSS 27.0 software was used for statistical analysis of experimental data. Statistical significance was determined using one-way ANOVA. Post hoc analysis was performed using the Tukey HSD test for multiple comparisons when ANOVA was applied. The significance level was set at *p* < 0.05. All analyses were carried out in triplicate.

## 3. Results and Discussion

### 3.1. Characterization of a-ChNCs and AOPE

**Figure 1:**
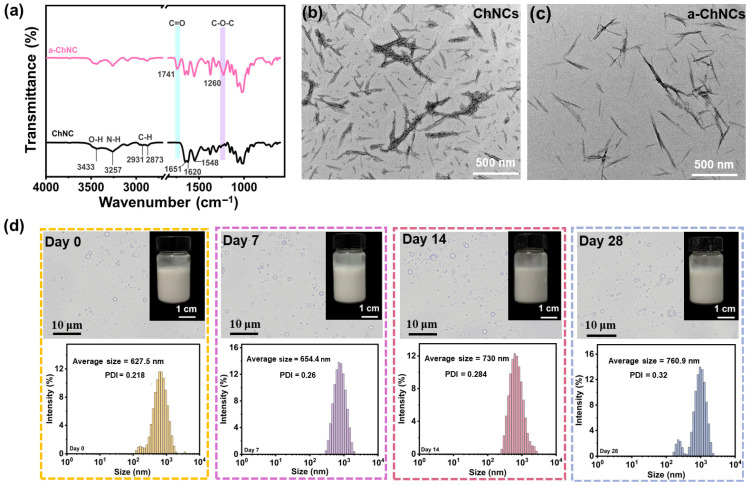
(**a**) FTIR spectra of the ChNCs and a-ChNCs. TEM images of (**b**) ChNCs and (**c**) a-ChNCs. (**d**) Optical microscopy image of AOPE and the droplet size distribution for different storage times.

The FTIR spectra depicted in Figure 1a demonstrate that a-ChNCs exhibited novel absorption bands at 1741 cm^−1^ and 1260 cm^−1^, corresponding to the stretching vibrations of carbonyl (C=O) and ether (C-O-C) functional groups, respectively. This observation, combined with the narrowing of the hydroxyl absorption peak at 3433 cm^−1^, suggests partial substitution of hydroxyl groups in the chitin backbone with acetyl groups [40]. The TEM of the nanocrystals (Figure 1b,c) revealed that ChNCs displayed a distinct, rigid, rod- or needle-like topological morphology. The integration of nanocrystals with high aspect ratios and rigid morphologies into the composite film can significantly enhance its mechanical strength and barrier properties [41]. a-ChNCs retained its original rod-like morphology; however, its edges became less distinct, and its diameter decreased. This phenomenon can be attributed to the hydrolysis of low-crystallinity regions on the ChNCs surface induced by acetic acid [42]. The particle size distribution curves of ChNCs and a-ChNCs are shown in Figure S4. The pristine ChNCs exhibit a relatively broad size distribution, mainly ranging from 100 to 200 nm with a PDI of 0.297, and a zeta potential (ζ) of 18.4 mV, indicating limited colloidal stability. In contrast, a-ChNCs display a more concentrated size distribution around 140 nm with a lower PDI of 0.217 and a significantly higher ζ potential of 38.2 mV. This suggests that acetylation of chitin hydroxyl groups enhances the positive surface charge, increases electrostatic repulsion between nanocrystals, reduces particle agglomeration, and thereby improves their dispersion stability in aqueous suspensions [43].

The XRD patterns of ChNCs and a-ChNCs are shown in Figure S5. Both samples exhibited diffraction peaks at 2θ = 9.2°, 19.2°, 20.7°, and 26.3°, corresponding to the (020), (110), (120), and (130) crystal planes of the typical α-chitin crystalline structure [44]. These results indicate that the intrinsic crystalline framework of chitin nanocrystals remained intact after surface acetylation. However, the crystallinity of a-ChNCs decreased from 87.3% to 78.5% compared with that of unmodified ChNCs. The reduction in diffraction peak intensity during the acetylation process can be attributed to the disruption of intra- and intermolecular hydrogen bonding caused by the modification of hydroxyl groups. Moreover, the modified nanocrystals exhibited reduced agglomeration, which can be attributed to the increased surface potential resulting from acetylation, leading to enhanced electrostatic repulsion and improved colloidal stability.

The encapsulation efficiency of OEO in the AOPE was determined to be 73.8%. The freshly prepared AOPE exhibited a particle size of 627.5 nm and a PDI of 0.218. This high uniformity likely contributes to consistent interfacial properties, effectively suppressing droplet aggregation and phase separation, thereby enhancing the kinetic stability of the emulsion system [45]. As shown in Figure 1d, the droplet size of AOPE gradually increased with prolonged storage time, without demulsification, indicating its excellent stability. The acetylation of hydroxyl groups on chitin nanocrystals enhanced electrostatic repulsion, thereby improving their dispersion in aqueous solutions and facilitating the formation of stable Pickering emulsions.

### 3.2. Characterization of GOP_X%_

**Figure 2:**
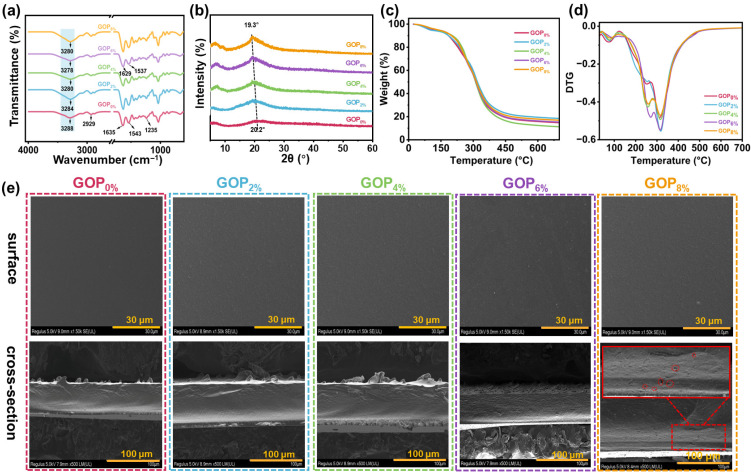
(**a**) FTIR spectra, (**b**) XRD patterns, (**c**) TGA curves, and (**d**) DTG curves of the composite films. (**e**) SEM images of composite films.

As shown in Figure 2a, the GOP_0%_ spectrum showed bands at 2921 cm^−1^ (asymmetric stretching of -CH_2_ groups, amide B), 1635 cm^−1^ (C=O stretching vibration of peptide bonds, amide I), 1543 cm^−1^ (C-N stretching and N-H bending vibrations, amide II), and 1238 cm^−1^ (C-N and N-H stretching vibrations coupled with -CH_2_ deformation, amide III) [46,47,48]. The broad absorption band at approximately 3288 cm^−1^ was attributed to the stretching vibrations of O-H and N-H groups (amide A of gelatin). The addition of AOPE induced a blue shift phenomenon in the stretching vibrations of the O-H and N-H groups in gelatin, indicating the formation of interfacial interactions (hydrogen bonds) between gelatin and AOPE. Additionally, the characteristic peaks for amide I (1635 cm^−1^) and amide II (1543 cm^−1^) shifted to lower wavenumbers (1629 cm^−1^ and 1537 cm^−1^, respectively). These shifts suggest the formation of intermolecular hydrogen bonds and potential electrostatic interactions between the amino groups of chitin and the carboxyl groups of gelatin [49].

The XRD patterns of GOP_X%_ are shown in Figure 2b. GOP_0%_ exhibited two distinct diffraction peaks at 2θ = 7.2° and 20.2°, corresponding to the triple-helical crystalline and amorphous free single-helical structures of gelatin, respectively [50]. Following the incorporation of AOPE, the intensity of the diffraction peak at 2θ = 7.2° was significantly enhanced, indicating that AOPE effectively promoted the formation of the triple-helical crystalline phase within the film matrix [51]. Additionally, the diffraction peak at 2θ = 20.2° showed increased intensity and shifted to 19.3° after adding AOPE. This effect was attributed to the presence of nanocrystals and the formation of additional hydrogen bonds between a-ChNCs and gelatin molecules, which collectively improved the crystallinity of the composite material [52,53].

Figure 2c,d display the TGA and derivative thermogravimetric (DTG) curves of GOP_X%_. TGA revealed that the thermal degradation profiles of the gelatin-based films featured three distinct stages of mass loss. The initial stage, observed between 50 °C and 130 °C, was attributed to the volatilization of free water molecules within the film matrix. The second decomposition stage, occurring between 130 °C and 275 °C, involved the volatilization of small molecules (e.g., OEO, glycerol) and partial thermal depolymerization of the gelatin matrix [54]. The final major mass loss stage, occurring between 290 °C and 350 °C, was attributed to the substantial thermal degradation of the gelatin, as well as the decomposition of a-ChNCs and other residual organic components [55,56]. The incorporation of AOPE led to an elevation in the second-stage decomposition temperature of the composite material, suggesting an improvement in thermal stability. This improvement is attributed to the increased crystallinity prompted by a-ChNC (Figure 2b).

Figure 2e shows the surface and cross-sectional morphologies of the composite films. The GOP_0%_ displayed a uniform and smooth surface morphology, along with a compact and continuous internal microstructure. Upon incorporating AOPE, surface roughness increased slightly; this effect intensified with higher emulsion content while maintaining overall structural uniformity and integrity. The film’s surface roughness likely resulted from emulsion droplet migration and partial aggregation during drying. The cross-sectional morphologies remained compact and slightly rough, with no visible cracks. However, at 8% AOPE content, small pores emerged in the cross-section. These pores were attributed to localized shrinkage caused by uneven evaporation rates of water and AOPE during film formation and drying [57].

### 3.3. Mechanical Properties

**Figure 3:**
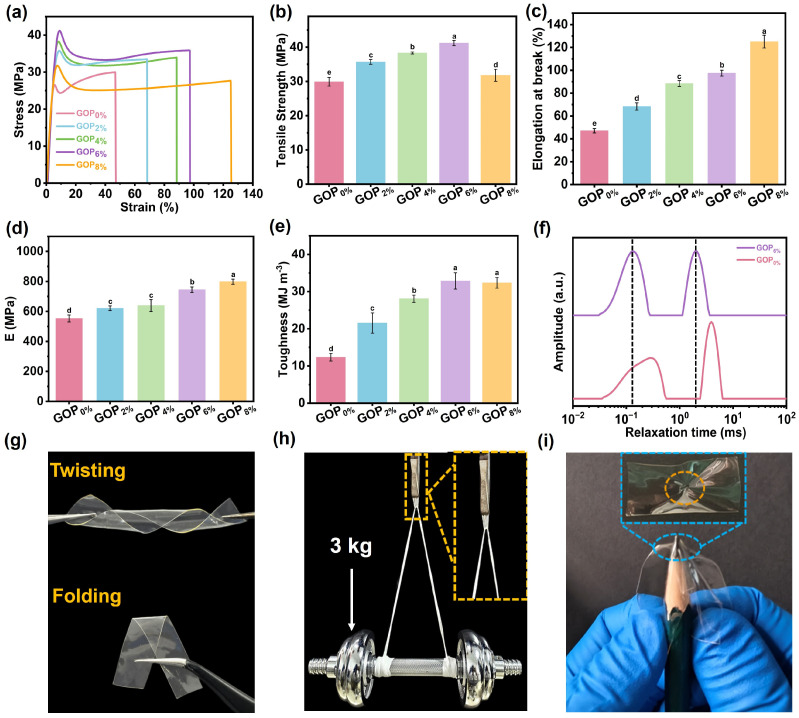
(**a**) Stress–strain curves, (**b**) tensile strength, (**c**) elongation at break, (**d**) Young’s modulus, and (**e**) toughness of composite films. (**f**) LF-NMR spectra of GOP_0%_ and GOP_6%_. (**g**) Twisting and folding images of the GOP_6%_ film. (**h**) Optical image showing GOP_6%_ film supporting 3 kg. (**i**) Puncture resistance of the GOP_6%_ film. Different letters on the bar indicate significant differences (*p* < 0.05).

The mechanical properties of the GOP_X%_ were evaluated using a stretching speed of 50 mm/min. Representative stress–strain curves are presented in Figure 3a. Key mechanical parameters, including maximum tensile stress, elongation at break, Young’s modulus, and toughness, are shown in Figure 3b–e. The maximum tensile strength and Young’s modulus increased from 29.9 MPa and 553.4 MPa (GOP_0%_) to 41.2 MPa and 744.9 MPa (GOP_6%_), respectively. The surface of a-ChNC is rich in hydroxyl groups, which can form hydrogen bonds with hydroxyl, amino, or carboxyl functional groups present on the molecular chains of gelatin. These interfacial interactions enhance the interfacial bonding between the nanocrystals and the gelatin matrix, thereby promoting the formation of a more robust three-dimensional network structure (Figure 3f) and contributing to improved mechanical strength [58]. In particular, the hydrogen bonds act as reversible physical cross-linkers that strengthen interfacial adhesion and restrict polymer chain mobility, forming a dense yet dynamic polymer network that enables concurrent enhancements in tensile strength, elongation, and toughness. It is widely recognized that dense physical cross-linking enhances polymer networks at the expense of ductility and toughness, reflecting the intrinsic trade-off between strength and ductility [59]. Remarkably, in this study, the elongation at break and toughness were increased from 47.1% and 12.3 MJ m^−3^ (GOP_0%_) to 97.5% and 32.9 MJ m^−3^ (GOP_6%_), achieving 107% and 167.5% enhancements, respectively. This breakthrough originates from the stress transfer and distribution mechanism mediated by interfacial interactions, which enables effective stress dissipation through cyclic hydrogen bond rupture-reformation processes within the network [60]. However, as the AOPE content was further increased, the maximum tensile strength of the GOP_8%_ decreased to 31.8 MPa. This decline is ascribed to the reduced mechanical integrity caused by excessive AOPE agglomeration within the composite matrix. Concurrently, the elongation at break of the GOP_8%_ increased. This behavior was hypothesized to result from the plasticizing effect of excess OEO, which weakened intermolecular interactions and enhanced ductility [8].

The prepared GOP_6%_ film exhibited high flexibility, as demonstrated in Figure 3g, allowing for arbitrary twisting and folding. As shown in Figure 3h, a 100 mg GOP_6%_ thin strip sample can lift a 3 kg barbell (30,000 times its weight), demonstrating exceptional mechanical robustness. Meanwhile, the GOP_6%_ film also exhibited exceptional puncture resistance, showing no signs of puncture damage (Figure 3i). Following the puncture test performed manually with a pencil, the sample exhibited good resistance to deformation, although slight wrinkles remained on the surface due to localized stress. This is primarily attributed to the ability of a-ChNC to transfer and dissipate stress throughout the composite material system. Furthermore, the dynamic network of reversible non-covalent bonds within the polymer matrix underwent dissociation-recombination cycles under load, enabling efficient energy dissipation and endowing the material with exceptional puncture resistance [60]. Figure 3g–i demonstrate that the GOP_6%_ film possesses exceptional mechanical robustness, flexibility, and puncture resistance, fully satisfying the key mechanical properties requirements for packaging materials.

### 3.4. Barrier Properties

**Figure 4:**
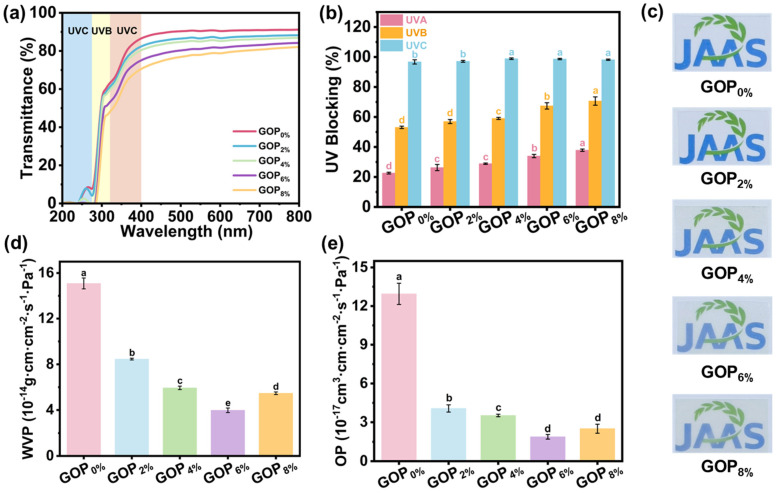
(**a**) UV-Vis spectra, (**b**) UVA, UVB and UVC blocking rates, (**c**) optical photographs, (**d**) water vapor permeability, and (**e**) oxygen permeability of the composite films. Different letters on the bar indicate significant differences (*p* < 0.05).

As shown in Figure 4a,b, the incorporation of AOPE markedly enhanced the UV-blocking performance. The UVC barrier increased from 96.8% to 98.2%, the UVB barrier increased from 53.1% to 70.7%, and the UVA barrier increased from 22.6% to 37.9%. Such improvement can be attributed to the following factors: firstly, phenolic compounds exhibit strong absorption in the UV region around 280 nm [61]; secondly, a-ChNCs exerts a light-scattering effect due to its nanoscale dimensions and large surface area, thereby improving the composite film’s UV-blocking performance [62]. Furthermore, visible light transmittance (380–780 nm) decreased progressively with higher AOPE content (Figure 4a,c). This reduction is primarily attributed to enhanced crystallinity within the composite film.

The water vapor permeability (WVP) and oxygen permeability (OP) of films are crucial for packaging applications, as they help prevent moisture uptake, inhibit oxidation, and delay spoilage [63]. As shown in Figure 4d,e, incorporating AOPE into GOP_X%_ films led to a pronounced decrease in both WVP and OP. The WVP value decreased from 1.51 × 10^−13^ g·cm·cm^−2^·s^−1^·Pa^−1^ (GOP_0%_) to 3.98 × 10^−14^ g·cm·cm^−2^·s^−1^·Pa^−1^ (GOP_6%_), corresponding to a 73.6% reduction, while the OP value decreased from 1.29 × 10^−16^ cm^3^·cm·cm^−2^·s^−1^·Pa^−1^ (GOP_0%_) to 1.89 × 10^−17^ cm^3^·cm·cm^−2^·s^−1^·Pa^−1^ (GOP_6%_), representing an 85.3% decrease. Compared with previously reported gelatin-based films (Table S1), the GOP_6%_ film exhibited markedly superior barrier performance. For instance, Zhu et al. reported that the incorporation of vanillin and Zn^2+^ into chitosan-gelatin films reduced the WVP from 2.19 × 10^−6^ to 1.33 × 10^−6^ g/(m·h·Pa) and the OP from 8.50 × 10^−7^ to 5.20 × 10^−7^ g/(m·h·Pa), corresponding to reductions of 39.2% and 38.8%, respectively [64]. Guo et al. found that incorporating 10 wt% organically modified montmorillonite into gelatin/cassava starch films decreased WVP and OP by 25% and 34%, while enhancing hydrophobicity and tensile strength [65]. The enhanced barrier properties can be attributed to two major factors: (i) the incorporation of AOPE increased the physical cross-linking density and network strength of GOP_X%_ (Figure 3f), thereby reducing the free volume and consequently decreasing the diffusion pathways of water vapor and oxygen molecules [66]; (ii) the uniform dispersion of AOPE within the gelatin matrix formed hydrophobic domains that hindered water permeation [24]. However, when the AOPE was added at 8%, both OP and WVP slightly declined, mainly due to AOPE agglomeration and OEO volatilization. These processes created pores within the film (Figure 2e), which in turn facilitated the diffusion of oxygen and water molecules.

### 3.5. Antibacterial and Antioxidant Properties

**Figure 5:**
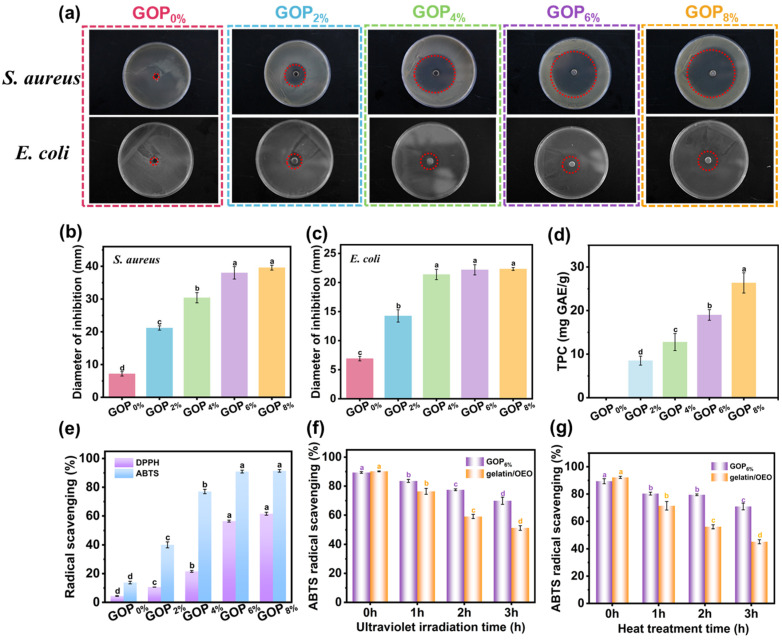
(**a**) Inhibition zones of GOP_X%_ against *S. aureus* and *E. coli* (red dashed circles representing the inhibition zones). Diameter of inhibition zones against (**b**) *S. aureus* and (**c**) *E. coli*. (**d**) Total phenol content and (**e**) radical scavenging of GOP_X%_. ABTS radical scavenging of GOP_6%_ and gelatin/OEO following UV (**f**) and heat (**g**) treatments. Different letters indicate significant differences (*p* < 0.05).

The antibacterial activity of active packaging films is crucial for inhibiting microbial growth, extending food shelf life, and ensuring safety. The antibacterial activity of composite films was evaluated using the agar diffusion method against *E. coli* and *S. aureus*. As shown in Figure 5a, the GOP_0%_ film exhibited no antibacterial activity, consistent with the inherent lack of antibacterial properties in gelatin. With increasing AOPE content, the inhibition zones became progressively larger. Specifically, the inhibition zone diameters for *E. coli* and *S. aureus* increased from 8.69 ± 1.06 mm and 21.16 ± 0.66 mm (GOP_2%_) to 22.32 ± 0.24 mm and 39.58 ± 0.74 mm (GOP_8%_, Figure 5b,c), respectively. Notably, all composite films demonstrated significantly larger inhibition zones against *S. aureus* (Gram-positive) than *E. coli* (Gram-negative), indicating superior antibacterial efficacy of OEO against Gram-positive bacteria. This discrepancy arises from structural differences in bacterial cell walls: *S. aureus* has a thick peptidoglycan layer with pores that allow phenolic compounds to penetrate, leading to membrane disruption and protein denaturation. In contrast, the outer membrane of *E. coli* is rich in lipopolysaccharide (LPS), which form a permeability barrier. This barrier restricts the entry of phenolic compounds such as thymol and carvacrol, thereby reducing antibacterial efficacy [67].

The polyphenolic compounds abundant in essential oils act as potent antioxidants and free radical scavengers, slowing oxidation processes. As shown in Figure 5d, the GOP_0%_ film exhibited no detectable total phenolic content (TPC). In contrast, TPC values for the GOP_2%_, GOP_4%_, GOP_6%_, and GOP_8%_ films increased to 8.51 ± 1.03, 12.76 ± 1.95, 18.99 ± 1.21, and 26.30 ± 2.32 mg GAE/g, respectively. As shown in Figure 5e, a positive correlation existed between the ABTS radical scavenging ability of the films and their TPC. The GOP_0%_ film, although lacking detectable phenolic compounds, still demonstrated a 13.8% radical scavenging rate, likely due to antioxidant peptides present in gelatin [48]. With the incorporation of AOPE, antioxidant activity was enhanced, increasing radical scavenging rates to 40.1%, 77.0%, 90.9%, and 91.4% for GOP_2%_, GOP_4%_, GOP_6%_, and GOP_8%_, respectively. This corresponds to increases of 190.5%, 457.9%, 558.6%, and 562.3% compared to GOP_0%_. The phenolic and terpenoid compounds in OEO contributed to the films’ antioxidant performance via radical scavenging, reduction, and singlet oxygen quenching [68]. Similarly, DPPH radical scavenging showed a comparable dose-dependent trend, though overall efficiency was slightly lower than that for ABTS. The GOP_0%_ film showed a scavenging rate of 4.4%, while the incorporation of AOPE significantly improved activity to 10.6%, 21.5%, 56.4%, and 61.5% for GOP_2%_, GOP_4%_, GOP_6%_, and GOP_8%_, respectively. The lower DPPH efficiency may result from differences in reaction kinetics and solubility between the two radical systems [67].

To evaluate the protective effect of the emulsion encapsulation structure on OEO, we compared the antioxidant activity of the GOP_6%_ film and the gelatin/OEO film under UV irradiation (Figure 5f) and heat treatment (Figure 5g). The results demonstrate that both UV radiation and heat treatment led to a significant reduction in the antioxidant activity of the films, with the extent of reduction increasing with prolonged exposure time. However, under identical conditions, the GOP_6%_ film consistently demonstrated retained antioxidant activity than the gelatin/OEO film, indicating that the emulsion encapsulation structure effectively protected OEO. The enhanced antioxidant retention of the GOP_6%_ film can be attributed to two synergistic mechanisms: (i) the hydrogen-bonded network also contributes to the retention of oregano essential oil by reducing the free volume and molecular mobility within the gelatin matrix, thereby limiting diffusion and volatility; (ii) the rigid a-ChNCs shell at the oil-water interface provides a physical barrier that protects the encapsulated oil from oxidation and thermal degradation [69].

### 3.6. Swelling Rate (SR) and Water Solubility (WS)

**Figure 6:**
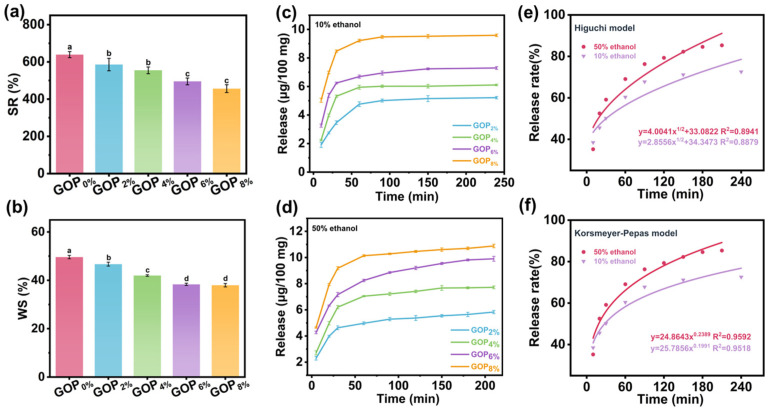
(**a**) Swelling rate and (**b**) water solubility of GOP_X%_. Release profiles of GOP_X%_ in (**c**) 10% ethanol and (**d**) 50% ethanol. (**e**,**f**) OEO release kinetic model fitting results (for GOP_6%_). Different letters on the bar indicate significant differences (*p* < 0.05).

As shown in Figure 6a, the swelling rate (SR) decreased significantly upon AOPE addition, which may be attributed to the enhanced intermolecular interactions and increased hydrophobicity imparted by AOPE. These effects reduce the availability of hydrophilic groups within the film matrix and consequently limit water uptake. The GOP_0%_ exhibited the highest WS (49.5 ± 0.7%), reflecting gelatin’s inherent hydrophilicity (Figure 6b). The WS values of the GOP_2%_, GOP_4%_, GOP_6%_, and GOP_8%_ films gradually decreased to 46.6 ± 0.8%, 41.9 ± 0.3%, 38.3 ± 0.4%, and 37.9 ± 0.7%, respectively. This reduction may be due to AOPE promoting the formation of a denser and less permeable film matrix [70]. Overall, these results indicate that AOPE effectively enhances the water resistance of gelatin-based films.

### 3.7. Release Characteristics of Composite Films

10% ethanol solution (representing hydrophilic foodstuffs) and 50% ethanol solution (representing semi-fatty foodstuffs) were selected as food simulants to evaluate the OEO release behavior of the films. The release of OEO from GOP_X%_ films into food simulants mainly involves two processes [71]: (1) water molecules are initially absorbed by the film surface and then gradually penetrate into the polymer network, causing the film matrix to swell; (2) the embedded OEO diffuses through the swollen network, migrates and dissolves in the external food simulant.

As shown in Figure 6c,d, the release profiles in both simulants displayed an initial rapid release phase followed by a slower, sustained release until equilibrium was reached. The OEO release reached equilibrium earlier in the 10% ethanol system due to its higher polarity and water content, which promote greater film swelling and thereby accelerate diffusion. Although the diffusion rate was faster in 10% ethanol, the total amount of OEO released was higher in the 50% ethanol system, owing to the greater solubility of OEO in ethanol that enhances its migration and dissolution at higher ethanol concentrations [72]. The release kinetics of OEO from the GOP_6%_ film were further analyzed using the Higuchi and Korsmeyer–Peppas models (Figure 6e,f). The Korsmeyer–Peppas model showed higher correlation coefficients (R^2^ = 0.9592 for 50% ethanol and 0.9518 for 10% ethanol) than the Higuchi model (R^2^ = 0.8941 for 50% ethanol and 0.8879 for 10% ethanol). The release exponent (*n*) values of 0.2389 and 0.1991 (<0.45) confirmed that the OEO release followed a Fickian diffusion mechanism [73].

### 3.8. Application of Films in Grass Carp Preservation

#### 3.8.1. pH Analysis

**Figure 7:**
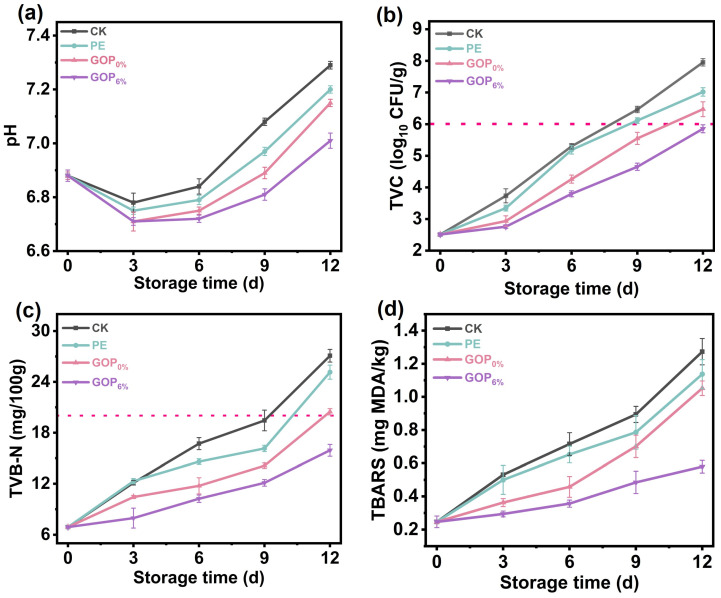
(**a**) pH, (**b**) TVC, (**c**) TVB-N, and (**d**) TBARS values of grass carp during storage.

Postmortem biochemical processes in fish muscle cause characteristic pH changes, which are universally accepted as critical indicators of fish freshness. As shown in Figure 7a, all samples exhibited an initial decrease in pH followed by a subsequent gradual increase under storage. The early decline (days 0–3) was attributed to lactic acid accumulation from post-mortem anaerobic glycolysis of muscle glycogen. Subsequently, the pH progressively increased, primarily due to microbial proteolysis and the formation of alkaline volatile amines [74]. On day 9, the pH of the CK group increased to 7.29 ± 0.01, significantly higher than that of the GOP_6%_ group, indicating that the active film effectively retarded pH elevation by inhibiting microbial proteolytic activity and thereby better preserving fish quality during storage.

#### 3.8.2. TVC Analysis

TVC is extensively utilized to assess the quality and safety of fish, with a value exceeding 6 log_10_ CFU/g typically indicating spoilage [75]. As shown in Figure 7b, the initial TVC of grass carp fillets was 2.51 ± 0.04 log_10_ CFU/g, demonstrating effective sterile handling during harvest and primary processing. During refrigerated storage, microbial proliferation progressively increased TVC values across all groups. On day 9, the TVC of the CK, PE, and GOP_0%_ groups all exceeded 6 log_10_ CFU/g, indicating significant deterioration of the fish. However, even on day 12, the TVC of the GOP_6%_ group remained below the 6 log_10_ CFU/g threshold. This prolonged microbial suppression is attributed to AOPE’s slow release of antibacterial phenolics, which disrupt bacterial membrane integrity and metabolic functions.

#### 3.8.3. TVB-N Analysis

As shown in Figure 7c, TVB-N in all groups progressively increased during storage, consistent with typical spoilage trends. On day 12, the TVB-N values of CK, PE, and GOP_0%_ groups exceeded the limit set by the Chinese standard GB 2733-2015 (20 mg/100 g) [76]. In contrast to the CK, PE, and GOP_0%_ groups, the GOP_6%_ group showed significantly lower TVB-N accumulation, suggesting effective inhibition of protein degradation by the AOPE-incorporated gelatin film. This preservation effect was due to the combined antibacterial and antioxidant properties of AOPE. Notably, after 12 days of storage, the TVB-N in the GOP_6%_ group was 15.93 ± 0.69 mg/100 g.

#### 3.8.4. TBARS Analysis

The degree of lipid oxidation, reflected by TBARS values, gradually increased with prolonged storage time (Figure 7d), which can be attributed to the accumulation of degradation products derived from lipid hydroperoxides and peroxides. The GOP_6%_ group demonstrated the most effective inhibition of lipid oxidation, outperforming the CK, PE, and GOP_0%_ groups. On day 12, the GOP_6%_ group showed a lower TBARS value of 0.57 ± 0.03 mg/kg. This enhanced preservation likely stems from synergistic mechanisms: (i) the enhanced oxygen barrier of GOP_6%_ film restricted oxygen penetration, thereby suppressing lipid peroxidation [77]; (ii) the incorporation of OEO endowed the film with strong free radical scavenging ability, effectively neutralizing reactive oxygen species (ROS) and interrupting the propagation of lipid radicals during storage [78].

#### 3.8.5. Protein Oxidation Analysis

**Figure 8:**
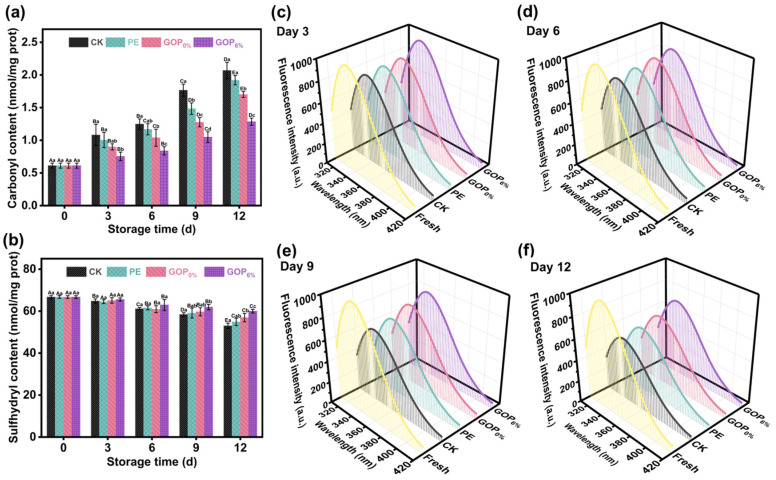
(**a**) Carbonyl and (**b**) total sulfhydryl content of grass carp fillets during storage. (**c**–**f**) Changes in myofibrillar protein fluorescence intensity during storage. Different capital letters indicate significant differences (*p* < 0.05) between groups, and different lowercase letters indicate significant differences within groups.

Carbonyl content is a key indicator for assessing the degree of protein oxidation. As shown in Figure 8a, the initial carbonyl content of myofibrillar proteins (MP) from fresh grass carp was 0.61 ± 0.04 nmol/mg prot. On day 12, the carbonyl content in the CK, PE, and GOP_0%_ groups had sharply increased to 2.06 ± 0.12, 1.92 ± 0.07, and 1.70 ± 0.04 nmol/mg prot, respectively, indicating a pronounced trend of oxidative deterioration. In contrast, the GOP_6%_ group exhibited a lower carbonyl level of 1.28 ± 0.06 nmol/mg prot, likely due to the antioxidant activity imparted by OEO. OEO likely inhibited oxidative modifications of amino acid residues (e.g., lysine, arginine, proline) by scavenging ROS [79]. Aldehyde by-products formed during lipid oxidation can react with nucleophilic sites on protein side chains, facilitating the accumulation of carbonyl content [80]. GOP_6%_ effectively inhibited lipid oxidation (Figure 7d), which may have reduced the levels of aldehyde by-products and thereby indirectly mitigated protein oxidation. This dual synergistic mechanism (free radical scavenging and lipid oxidation suppression) effectively mitigated oxidative damage to MP in grass carp fillets during storage.

Sulfhydryl groups (-SH), primarily derived from cysteine residues, are essential functional groups in MP, playing a key role in maintaining its tertiary structure and functional integrity [81]. During oxidation, -SH groups are readily oxidized to form disulfide bonds, leading to conformational changes that affect the physicochemical properties of proteins [82]. As shown in Figure 8b, the total sulfhydryl (T-SH) content in all groups progressively declined during storage. In the CK group, T-SH significantly declined from 66.73 ± 0.65 to 53.02 ± 1.27 nmol/mg prot (a 21.2% reduction), demonstrating substantial oxidative depletion of sulfhydryl groups. In contrast, the GOP_6%_ group showed a slower decrease, maintaining a T-SH level of 60.01 ± 0.90 nmol/mg prot on day 12 (only a 10.1% reduction). This protective effect may be attributed to the strong free radical scavenging capacity of GOP_6%_, which mitigated hydroxyl radical attacks and inhibited MDA formation, thereby retarding protein oxidation.

The tertiary structure of MP can be evaluated using intrinsic fluorescence, as it reflects changes in the microenvironment surrounding tryptophan residues [83]. Tryptophan residues are typically buried within the hydrophobic regions of the native protein; however, oxidative damage can cause protein unfolding and expose these residues, leading to decreased fluorescence intensity. As shown in Figure 8c–f, the fluorescence emission spectra of MP during storage exhibited characteristic tryptophan emission peaks in the 320–340 nm range, with peak intensity progressively declining over time. This trend indicates gradual tertiary structural disruption and increased exposure of tryptophan residues to a polar environment. On day 9, a marked decline in fluorescence intensity was observed in the CK and PE groups, likely due to severe oxidative damage and consequent conformational instability [84]. In contrast, the GOP_6%_ group maintained the highest fluorescence intensity throughout the storage period and exhibited the smallest change in surface hydrophobicity (Figure S6). These results demonstrate that GOP_6%_ effectively mitigated oxidative damage and preserved the conformational stability of MP.

#### 3.8.6. Texture Analysis

**Figure 9:**
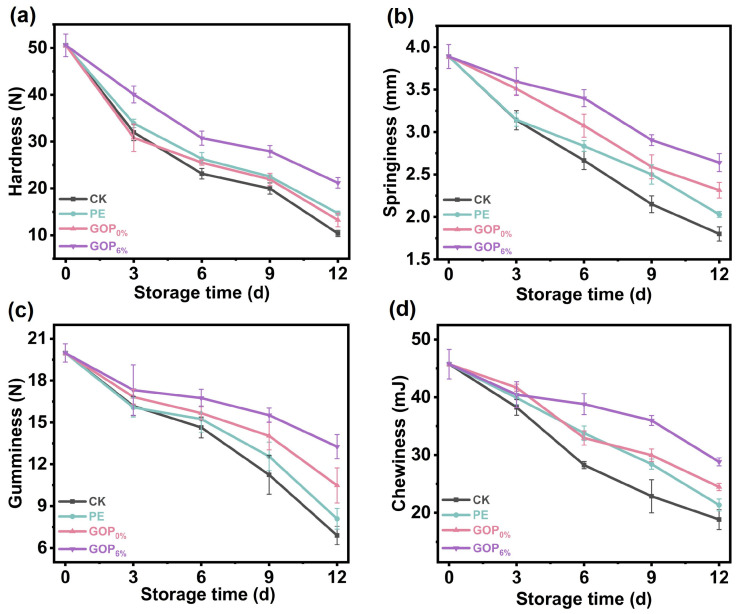
(**a**) Hardness, (**b**) springiness, (**c**) gumminess, and (**d**) chewiness of grass carp during storage.

Texture parameters serve as critical indicators for assessing the quality characteristics of foods. As shown in Figure 9, the CK group experienced the most pronounced texture degradation during storage. On day 12, the CK group exhibited a marked reduction in texture parameters: hardness decreased from 50.5 ± 2.41 N to 10.4 ± 0.67 N; springiness decreased from 3.89 ± 0.14 mm to 1.80 ± 0.09 mm; gumminess decreased from 19.98 ± 0.65 N to 6.90 ± 0.64 N; and chewiness decreased from 45.73 ± 2.55 mJ to 18.80 ± 1.73 mJ. These substantial decreases indicate severe muscle structure breakdown due to enzymatic proteolysis and microbial activity [85]. In contrast, the GOP_6%_ group effectively preserved textural properties throughout storage, consistently maintaining superior performance compared to the CK, PE, and GOP_0%_ groups, with differences becoming more pronounced towards the end of storage. This enhanced performance can be attributed to AOPE, which improves oxygen and moisture barrier performance, reduces oxidative damage to muscle proteins, and suppresses microbial proliferation [86].

Generally, GOP_6%_ exhibited favorable mechanical properties along with strong antioxidant and antimicrobial activities, which effectively retarded quality deterioration in grass carp during storage. These advantages are closely related to the incorporation of a-ChNCs, whose preparation is both simple and economically viable, relying on inexpensive reagents such as acetic anhydride and sulfuric acid. Moreover, the small loading of a-ChNCs in the film imposes minimal impact on overall cost. The considerable improvements in material performance and stability therefore provide a favorable cost-to-performance ratio, supporting the scalability and commercial potential of this approach.

## 4. Conclusions

In this study, a-ChNCs were selected as the stabilizer to successfully prepare OEO Pickering emulsions (AOPE). By leveraging the interfacial interactions between a-ChNCs and gelatin molecules, multifunctional AOPE/gelatin composite films were constructed. The resulting GOP_6%_ film exhibited outstanding comprehensive properties: elongation at break reached 97.5% (2.1 times of GOP_0%_), and toughness achieved 32.9 MJ m^−3^ (2.7 times of GOP_0%_); WVP decreased to 3.98 × 10^−14^ g·cm·cm^−2^·s^−1^·Pa^−1^ (26.4% of GOP_0%_), and OP reduced to 1.89 × 10^−17^ cm^3^·cm·cm^−2^·s^−1^·Pa^−1^ (14.7% of GOP_0%_). An in-depth analysis of the reinforcement mechanism revealed the critical role of a multiscale synergistic effect: on one hand, the interfacial interactions between gelatin and a-ChNCs enhanced the physical crosslinking density and strength of the polymer network, facilitated efficient stress transfer and distribution, and reduced polymer free volume; on the other hand, the hydrophobic microdomains formed by the AOPE effectively impeded water molecule diffusion. The AOPE structure also improved OEO stability and imparted significant antibacterial and antioxidant activities to the composite film. The GOP_6%_ film effectively inhibited the growth of foodborne pathogens and dominant spoilage bacteria in fish, thereby delaying quality deterioration in grass carp fillets during storage. This exceptional combination of high-performance properties positions the GOP_6%_ film as a highly promising candidate for eco-friendly active food packaging applications and provides a novel design strategy for future food packaging materials.

## Data Availability

The original contributions presented in this study are included in the article/Supplementary Materials. Further inquiries can be directed to the corresponding author.

## Supplementary Materials

The following supporting information can be downloaded at: https://www.mdpi.com/article/10.3390/foods14223978/s1, Figure S1. Oregano Essential Oil Standard Curve; Figure S2. Gallic acid standard curve for determination of total phenolic content (TPC); Figure S3. Standard curve of malondialdehyde (MDA) used for quantification of MDA content in samples; Figure S4. Particle size distribution curve of the (a) ChNCs and (b) a-ChNCs; Figure S5. XRD pattern of the ChNCs and a-ChNCs; Figure S6. Surface hydrophobicity of fish myofibrillar proteins during storage; Figure S7. The optical micrograph of the GOP_6%_ film-forming solution. Table S1. GOP_6%_ property values for similar gelatin-based active films. References [87,88,89] are cited in the Supplementary Materials.

## Abbreviations

The following abbreviations are used in this manuscript:

AOPE	Oregano essential oil Pickering emulsion
GOP_X%_	Gelatin composite film
OEO	Oregano essential oil
ChNCs	Chitin nanocrystals
PDI	Polydispersity index
WVP	Water vapor permeability
OP	Oxygen permeability
SR	Swelling rate
WS	Water solubility
TPC	Total phenolic content
TVC	Total viable count
MDA	Malondialdehyde
TVB-N	Total volatile basic nitrogen
TBARS	Thiobarbituric acid-reactive substances
ROS	Reactive oxygen species
MP	Myofibrillar proteins
-SH	Sulfhydryl groups
