# Supplementary material for: Interfacial Engineering of High-Performance Pickering Emulsion–Gelatin Composite Films for Active Packaging
Source: Foods. 2025 Nov 20;14(22):3978. doi: 10.3390/foods14223978 (PMC12652791; doi:10.3390/foods14223978)
Supplement: Supplementary file 1 [file foods-14-03978-s001.zip › foods-3951253-supplementary.pdf]

Supplementary Materials

Interfacial Engineering of High-Performance Pickering Emulsion–Gelatin Composite Films for Active Packaging

Jia Kan [1,2,3], MingZhu Li [1,2,3], MengHuan Liu [1,2,3], Ning Jiang [1,2,3,*], ZeFeng Yue [1,2], Hao Yu [1,2], RongXue Sun [1,2], QianYuan Liu [1,2], SaiKun Pan [3] and Cheng Wang [1,2,*]

[1] Institute of Agricultural Products Processing, Jiangsu Academy of Agricultural Sciences, Nanjing 210014, China; 17305263586@163.com (J.K.); m19741895219@163.com (M.L.); 17397495371@163.com (M.L.); yzf6782000@163.com (Z.Y.); 17855530610@163.com (H.Y.); sunrongxue187@163.com (R.S.); liu.qianyuan@foxmail.com (Q.L.)

[2] Integrated Scientific Research Base for Preservation, Storage and Processing Technology of Aquatic Products of the Ministry of Agriculture and Rural Affairs, Nanjing 210014, China

[3] College of Marine Food and Bioengineering, Jiangsu Ocean University, Lianyungang 222000, China; pskgx@163.com

* Correspondence: jaas_jiangning@163.com (N.J.); wangcheng@jaas.ac.cn (C.W.)

Supplementary figures

Standard curve equations:

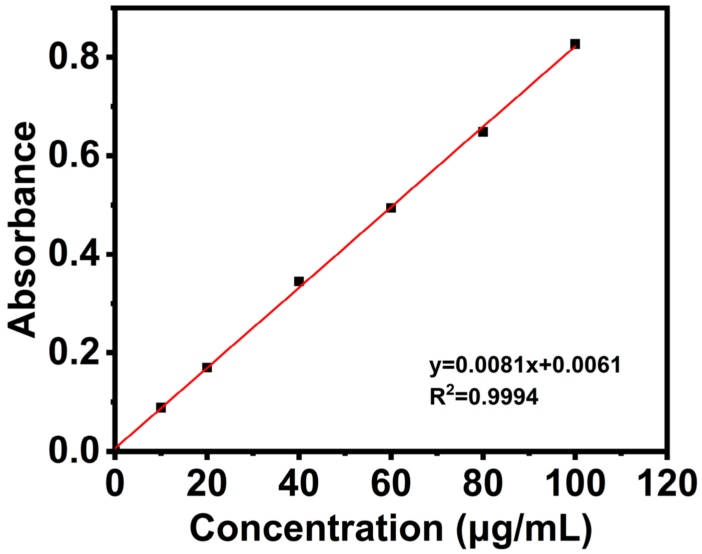

Figure S1. Oregano Essential Oil Standard Curve.

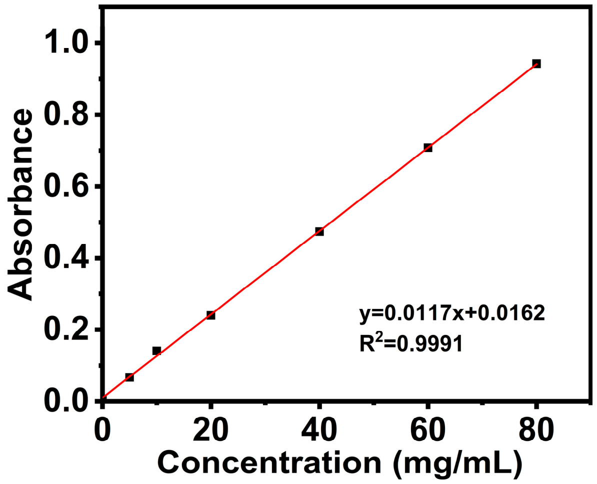

Figure S2. Gallic acid standard curve for determination of total phenolic content (TPC).

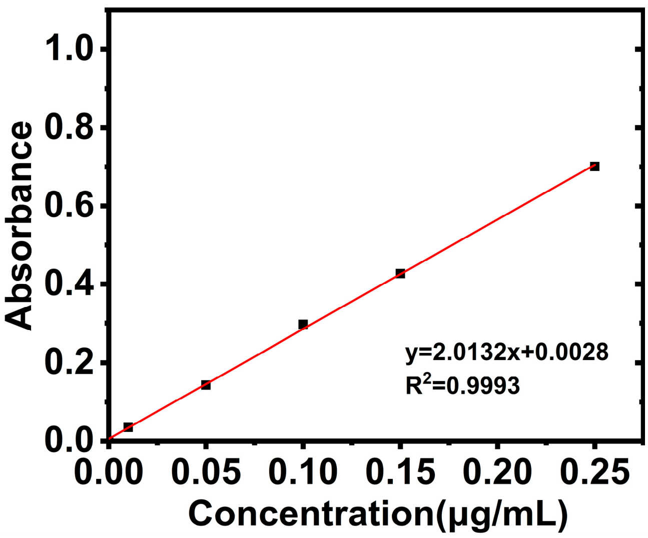

Figure S3. Standard curve of malondialdehyde (MDA) used for quantification of MDA content in samples.

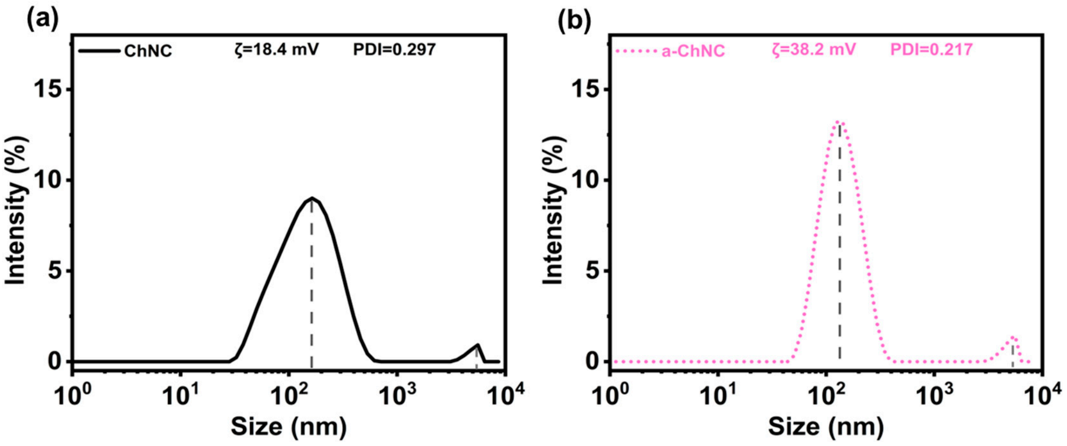

Figure S4. Particle size distribution curve of the (**a**) ChNCs and (**b**)a-ChNCs.

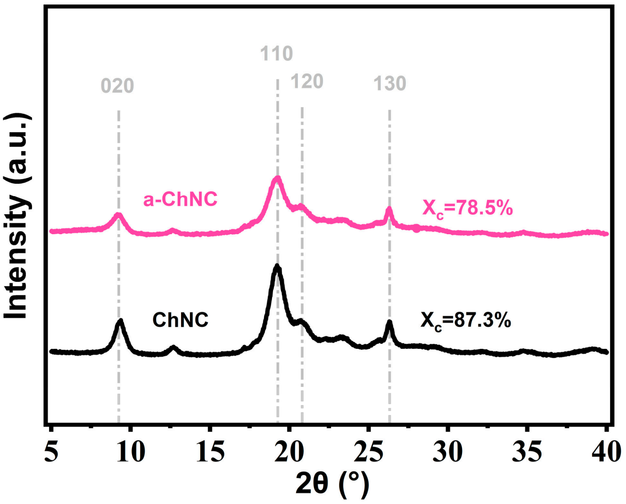

Figure S5. XRD pattern of the ChNCs and a-ChNCs.

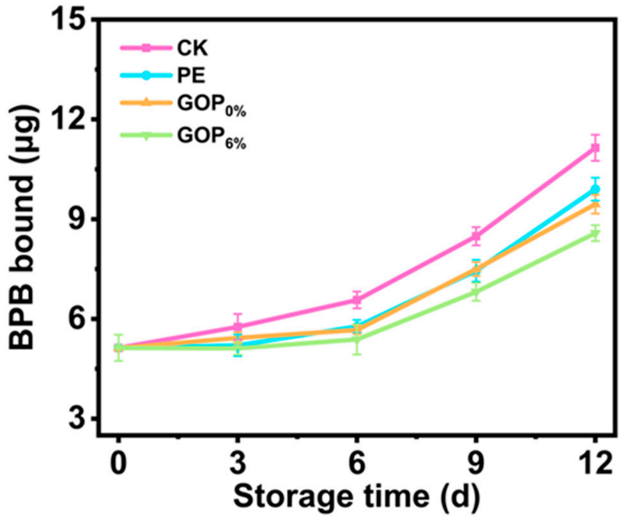

Figure S6. Surface hydrophobicity of fish myofibrillar proteins during storage.

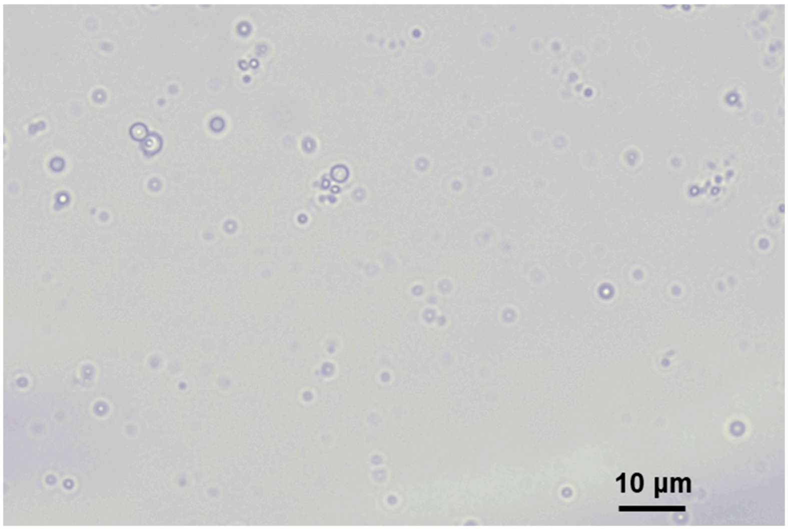

Figure S7. The optical micrograph of the GOP6% film-forming solution.

Table S1. GOP6% properties values for similar gelatin-based active films

Sample name	TS (MPa)	EB (%)	OP (10^{-17}g·cm^3·cm^{-2}·S^{-1}·Pa^{-1})	WVP （10^{-14}g·cm·cm^{-2}·S^{-1}·Pa^{-1}）	ABTS radical scavenging (%)
GOP6%	41.2 ± 0.78	97.5 ± 2.5	1.89 ± 0.17	3.98 ± 0.19	90.9 ± 1.0
Gel/Zn-MOF1% [1]	88.0 ± 3.6	6.8 ± 1.1	/	7.7 ± 0.1	84.4 ± 1.6
SG/20RES [2]	5. 45± 0.12	55.86 ± 1.5	8.2 ± 0.7	3.61 ± 0.16	81.12 ± 0.6
GDG-3 [3]	49.4 ± 4.5	55.7 ± 9.8	2.4 ± 1.6	6.7 ± 0.75	85.3 ± 0.2
OMMT10/GC [4]	8.54 ± 021	27.39 ± 2.58	/	2.46 ± 0.09	/

References:

1. Riahi, Z.; Hong, S.J.; Rhim, J.-W.; Shin, G.H.; Kim, J.T. High-performance multifunctional gelatin-based films engineered with metal-organic frameworks for active food packaging applications. *Food Hydrocolloids* **2023**, *144*, 108984, doi:https://doi.org/10.1016/j.foodhyd.2023.108984.
2. Wu, H.; Li, T.; Peng, L.; Wang, J.; Lei, Y.; Li, S.; Li, Q.; Yuan, X.; Zhou, M.; Zhang, Z. Development and characterization of antioxidant composite films based on starch and gelatin incorporating resveratrol fabricated by extrusion compression moulding. *Food Hydrocolloids* **2023**, *139*, 108509, doi:https://doi.org/10.1016/j.foodhyd.2023.108509.
3. Khin, M.N.; Easdani, M.; Aziz, T.; Shami, A.; Alharbi, N.K.; Al-Asmari, F.; Lin, L. Schiff's base crosslinked gelatin-dialdehyde cellulose film with gallic acid for improved water resistance and antimicrobial properties. *Food Hydrocolloids* **2025**, *166*, 111331, doi:https://doi.org/10.1016/j.foodhyd.2025.111331.
4. Guo, L.; Li, C. Preparation of organically modified montmorillonite reinforced gelatin/cassava starch film and its application in broccoli florets preservation. *Food Packag. Shelf Life* **2025**, *52*, 101604, doi:https://doi.org/10.1016/j.fpsl.2025.101604.
